# Incidence and Survival of Patients with Small Intestinal Neuroendocrine Tumours in a Danish NET Center

**DOI:** 10.1100/2012/206350

**Published:** 2012-11-28

**Authors:** Lise Brehm Hoej, Karen Marie Nykjær, Henning Gronbaek

**Affiliations:** Department of Medicine V (Hepatology and Gastroenterology), Aarhus University Hospital, 8000 Aarhus, Denmark

## Abstract

*Introduction*. Small intestinal neuroendocrine tumours (NETs) have increased in incidence during the past decades. In recent years, new promising treatment modalities have been introduced. The aim of the present study was to characterize and compare patients with small intestinal NET seen in the periods 1994–2003 (group 1) and 2004–2011 (group 2) to demonstrate changes in incidence and survival in the two time periods. *Patients and Methods*. There were 52 NET patients in group 1 and 109 patients in group 2. *Results*. The incidence of small intestinal NET was 0.3/100.000/year in period 1 and 0.7/100.000/year in period 2. There was no difference in median chromogranin A levels (8709 versus 2381 pmol/L, *P* = 0.107), presence of liver metastases (56% versus 44%), clinical symptoms (flushing/diarrhea), or Ki67 index (2% versus 2%), between the two time periods. The 5-year survival rate in the two time periods was similar, 64.7%, and 77.0%, respectively, (*P* = 0.23). *Conclusion*. We observed an increase in the incidence of small intestinal NET during the period from January 1994 to December 2011, but we were not able to demonstrate an improved survival during the same time period.

## 1. Introduction

Neuroendocrine tumours (NETs) develop from cells of the diffuse neuroendocrine system [1]. NETs have been considered to occur quite rarely; however, the incidence of NETs is increasing, and according to the North American Surveillance, Epidemiology and End Results (SEER) Programme, the incidence increased from 1.1/100.000 per year in 1973 to 5.3/100.000 per year in 2004 [2]. One of the most common locations of neuroendocrine tumours is the small intestine with the primary tumour most often located in the distal ileum. The annual incidence of small intestinal NET is approximately 0.8/100.000 [3]. This is an increase by 460% over the last 30 years [4]. 

Due to the rarity and complexity of the disease, diagnosis and treatment of patients with neuroendocrine tumours must be centralised in specialised NET centers with the capacity of a multidisciplinary approach. Department of Medicine V (Hepatology and Gastroenterology), Aarhus University Hospital, Denmark, has since 1994 been responsible for NET patients in the western part of Denmark with a population of approximately 1.9 million. Due to new treatment modalities with life-prolonging potential, the prevalence of NET patients has increased. However, there are few data to demonstrate a change in survival over time. 

A clinical database including all patients with neuroendocrine tumours was initiated in 2003 and expanded and updated in 2011 at Aarhus NET center, Aarhus University Hospital. In 2007, we reported on patients with midgut carcinoid tumours seen at our department in the period from January 1994 to June 2003 [5]. 

The aim of the present study was to characterize patients with neuroendocrine tumours of the small intestine referred to our NET center in the period from January 1994 to June 2003 and to compare these with patients seen in the period July 2003 to December 2011. Further, we wanted to investigate the incidence of small intestinal NET in these two well-defined time periods and to compare survival in these two cohorts.

## 2. Patients and Methods

### 2.1. Patients

In the period between January 1994 and June 2003, 52 patients with histopathologically verified small intestinal NET were referred for further diagnosis and treatment. In the period between July 2003 and December 2011, 109 histopathologically verified small intestinal NET patients were seen at the department. Patients from these two time periods will be referred to as group 1 and group 2, respectively. Patients with unknown primary tumour were excluded from the study.

The study was approved by the Danish Data Protection Agency.

### 2.2. Data Collection

We retrospectively collected data from the patients medical records into a specially designed NET database. The data comprised basic patient information as well as information from medical consultations. Basic patient information included gender, date of birth, date of diagnosis, duration of symptoms prior to diagnosis, date of first visit in the NET center, primary tumour localisation, treatment prior to referral, and date of death. Information from medical consultations included clinical symptoms, histopathology, biochemistry, imaging results, and treatment. 

### 2.3. Statistics

Statistical analysis was performed using SPSS 18.0. Unless otherwise specified, values are expressed as medians (range) or percentages when appropriate. We used Mann-Whitney test in between groups and chi-square for categorical variables. *P* values are two sided and were considered significant if less than 0.05. The Kaplan-Meier method was used for survival analysis and the log-rank test for comparison of groups.

## 3. Results

In the period between January 1994 and June 2003, the annual incidence of histopathologically verified small intestinal NET was 0.3/100.000/year, and in the period between July 2003 and December 2011, the incidence increased to 0.7/100.000/year. We assumed that the population in the NET center catchment area was stable around 1.9 million people.

Demographic and clinical data for group 1 and group 2 are shown in Table 1. There were no differences regarding gender, age, and height at diagnosis. Patients in group 1 had a significantly lower body weight than patients in group 2.

Group 1 patients reported a significantly longer duration of symptoms prior to tumour diagnosis than did patients in group 2. We found no statistically significant differences in frequency of abdominal pain or symptoms related to the carcinoid syndrome, for example, diarrhea, flushing, or bronchial constriction at referral between the two groups.

As shown in Table 2, the primary tumour size did not differ significantly between the two groups. The median Ki67 index was similar and low (2%) in both patient groups. 

A significantly higher number of patients in group 1 than in group 2 presented with carcinomatosis at referral; however, more patients in group 2 had lymph-node metastases at referral. There was no difference between the groups in the incidence of liver metastases, bone metastases, or metastases to other tissues at the time of referral. Approximately half of patients in both groups presented with liver metastases. Only a minor proportion of patients had bone metastases (3–5%) and metastases to other tissues (6-7%).

The two groups did not differ significantly with regards to plasma CgA levels at referral to the department; however, urine 5-HIAA was higher in group 1 than in group 2.

Table 3 shows the different imaging modalities used as diagnostic procedures at referral to the NET center. The majority of patients in both groups had an octreotide receptor scintigraphy, for group 2 patients most often combined with contrast-enhanced CT scan. The number of patients receiving a CT scan increased during the observation period. Only a minor proportion of patients in both groups had an MRI scan performed, less than 5%. None of the patients in group 1 had a PET scan performed at referral, while a higher proportion had PET scan (Gallium-Dotanoc PET-CT, F-DOPA PET-CT) in group 2.

Table 4 shows the different treatment modalities used in the treatment of the patients in the two groups. We found no statistically significant difference in the proportion of patients that within the first few months after diagnosis underwent surgery with the removal of their primary tumour and metastases. However, of patients with surgery, significantly more patients in group 2 were radically operated compared to group 1.

During the followup period patients in both groups received a number of different treatment modalities. Significantly more patients in group 1 were treated with interferon, while significantly more patients in group 2 were treated with radiofrequency ablation and embolisation. Significantly more patients in group 2 underwent new surgery in the followup period. We observed no difference between the two groups with regards to the proportion of patients treated with somatostatin analogues, radionuclide treatment, systemic chemotherapy, and temozolomide.

**Figure 1:**
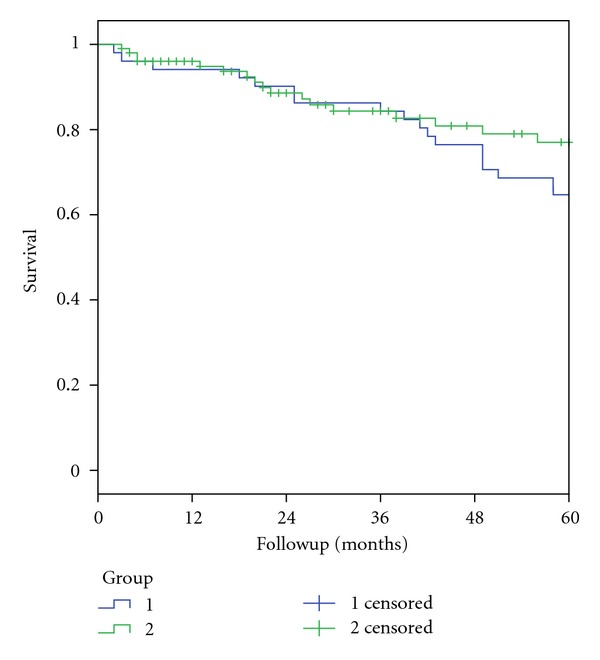
5-year survival rate for patients seen in the two time periods January 1994–June 2003 (Group 1) and July 2003–December 2011 (Group 2).

Figure 1 depicts the 5-year survival rates for the two groups. There was no difference in 5-year survival between the two groups (in group 1 64.7% ± 6.7% compared to 77.0% ± 5.1% in group 2, *P* = 0.23). However, there was not complete 5-year followup for all group 2 patients.

**Figure 2:**
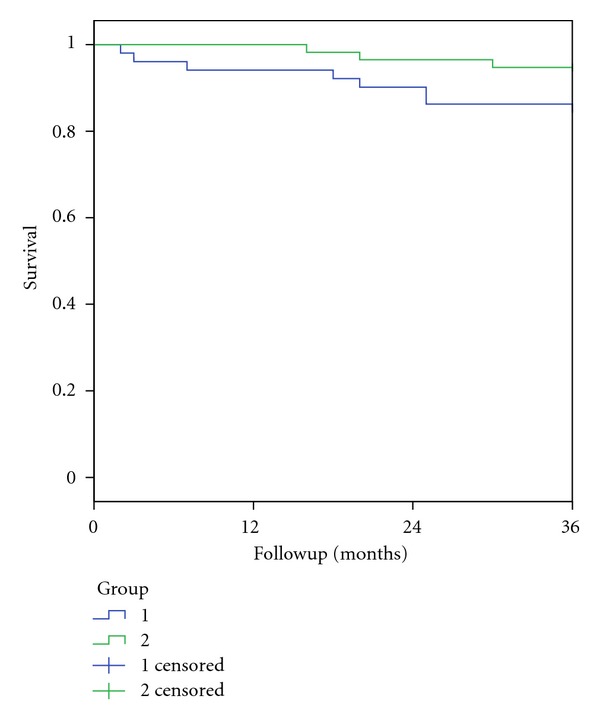
3-year survival rates for patients seen in the two time periods from January 1994 to June 2003 (Group 1) and from July 2003 to December 2011 (Group 2).

We therefore evaluated the 3-year survival rates for patients in the two groups with complete followup of a minimum of three years (Figure 2). There was a weak trend towards improved 3-year survival between the two groups (group 1: 86.3% ± 4.8%, group 2: 94.7% ± 3%, *P* = 0.125).

**Figure 3:**
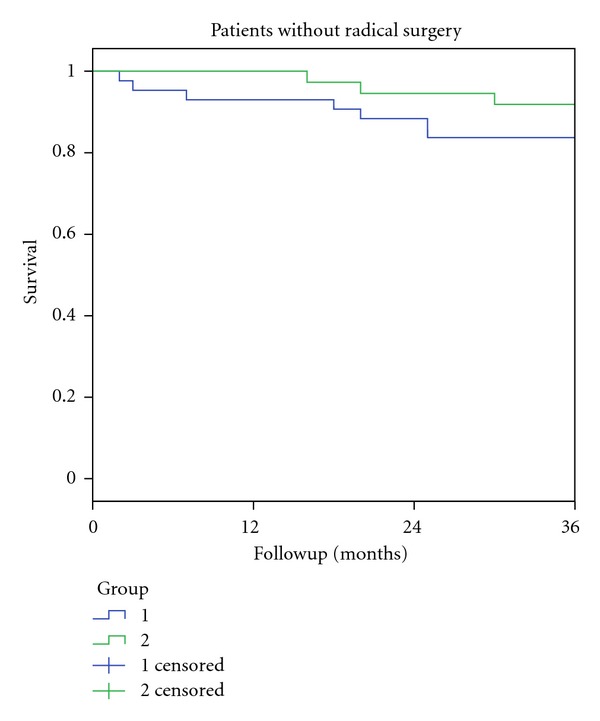
3-year survival rates for patients without radical surgery seen in the two time periods from January 1994 to June 2003 (Group 1) and from July 2003 to December 2011 (Group 2).

Figure 3 shows 3-year survival rates for patients without radical surgery in group 1 and group 2 with complete followup for at least three years. There was no significant difference in 3-year survival between the two groups (83.7% ± 5.6% in group 1 compared to 91.9% ± 4.5% in group 2, *P* = 0.26). For both groups, 3-year survival for patients with radical removal of all tumour tissues was 100%.

**Figure 4:**
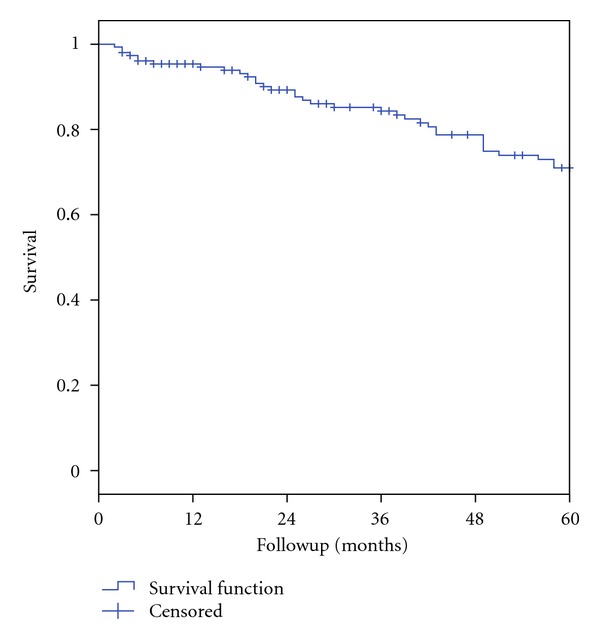
5-year survival rate for all patients with small intestinal NET seen in the period from January 1994 to December 2011.

Figure 4 shows the 5-year survival rate for all patients seen in the period between January 1994 and December 2011 with a five-year survival rate of 71.0% ± 4.2%.

**Figure 5:**
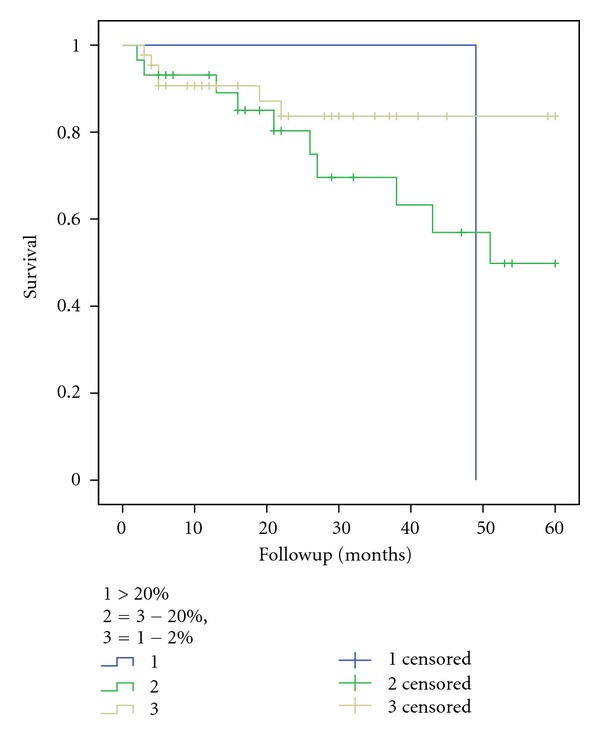
5-year survival rates according to Ki-67 index for all patients.

We investigated Ki67 index as a prognostic marker of survival for all patients (Figure 5). Tumours with low Ki67 index were associated with a better survival compared to tumours with higher Ki67 index. However, this finding was not statistically significant (*P* = 0.14).

## 4. Discussion

We present data regarding patients with small intestinal NET from a Danish NET center. The main finding of the present study was an increase in the incidence of small intestinal NET in the period from January 1994 to December 2011. However, we could not demonstrate a significant change in survival between the two time periods, which probably reflects the relatively short followup period. 

The demonstrated increase in incidence of small intestinal NET is in line with findings in other studies [2, 3, 6] with an incidence of approximately 0.6–0.8/100.000 per year. The observed increase in incidence in this study may partly be attributed to an increase in the rate of referral from local hospitals to our NET center. During the last two decades, there has been increased focus on referring all NET patients to specialised NET centers for optimal treatment and followup. Therefore, it is likely that more patients diagnosed with small intestinal NET in the second time period were referred to the NET center. However, the increase in incidence may also in part be explained by increased clinical awareness of the disease along with improved pathology investigations and imaging techniques. However, a true increase in the incidence of small intestinal NET is likely to exist too [7]. This may be supported by the fact that clinical characteristics of the patients were similar in the two time periods regarding tumour stage, presence of metastases, and so forth. However, we cannot exclude that some patients with complete removal of all tumour tissue at a local hospital were not referred. This could bias our results and underestimate the incidence of small intestinal NET and the survival rates due to the more favourable prognosis for radically operated patients.

Patients seen in the two time periods were similar, but more patients in group 1 had carcinomatosis at referral, while more patients in group 2 had lymph-node metastases at referral. The change in imaging techniques may explain the increased number of patients with lymph-node metastases due to the higher sensitivity of PET imaging used more frequently for group 2 patients. However, the groups did not differ with regards to the incidence of liver metastases and are therefore considered comparable in terms of disease stage at the time of referral. The 3- and 5-year survival rates did not differ considerably between the two groups despite the fact that more patients in group 2 had a successful surgery and a higher number of patients were treated with RFA. Radionuclide treatment was initiated in 2004 as a second- or third-line therapy and thus used late in the clinical course and has shown promising effects in several studies [8–10]. The same proportion of patients had radionuclide treatment, and it is possible that the effect of radionuclide treatment on the survival of our patients will appear after a longer period of followup, relevant for group 2 patients. 

The overall 5-year survival rate of all small intestinal NET patients in the two time periods was 71%. Other studies found 5-year survival rates of 60.5%–75% [11–15]. Direct comparison of survival rates is, however, difficult due to differences in tumour classification and patient cohorts. Some studies [12] report on midgut carcinoid tumours; which is a formerly used term comprising tumours from the distal duodenum to the midtransverse colon and so may also encompass tumours originating in the appendix. Appendiceal NETs have an excellent prognosis with reported disease-specific five-year survival rates of 89%–95% [13, 16, 17]. Survival rates for midgut carcinoid tumours as a group are therefore likely to be higher than the specific survival rates for patients with true small intestinal NET. 

Bergestuen et al. [14] compared the overall 5-year survival in their group of patients (72%) with the expected survival in the general population and observed a relative 5-year survival rate of 78%. 

In groups 1 and 2, 80.8% and 75.2% of patients, respectively, had a primary surgery performed after the initial evaluation. Of these patients, 19% in group 1 and 56% in group 2 were considered macroscopically radically operated. Patients in both groups with macroscopically radical surgery performed had a higher 5-year survival rate compared to patients in the same group without radical surgery performed. This observation is in line with the finding of Pape et al., where patients with radical tumour resection had a more favourable prognosis compared to the whole group of patients [15]. A similar observation was made by Hellman et al. [18]. 

This study only comprised patients with true primary tumour in the small intestine, and we excluded patients with unknown primary tumour. However, in some cases of NET of unknown origin, the primary tumour is later identified in the small intestine [7]. Having disseminated disease at the time of diagnosis, these patients have a poorer prognosis than the overall group of patients with small intestinal NET [13, 14, 19]. Consequently, the true survival rate for all the patients with small intestinal NET may be overestimated in this study.

In order to markedly improve the prognosis for patients with NET, the patients must be diagnosed at an earlier stage of the disease and before the tumour has disseminated. This is a challenge since most small intestinal NETs are nonfunctioning [20], and symptoms caused by nondisseminated tumours are often lacking or are vague and uncharacteristic [7]. Increased focus on NET as a possible cause of intermittent or persistent abdominal discomfort or diarrhea in combination with improved imaging techniques may lead to earlier diagnosis.

## 5. Conclusion

We observed an increase in the incidence of small intestinal NET in the period from January 1994 to December 2011, which may reflect a true increase in incidence however, increased focus and better imaging techniques may also be involved. We could not demonstrate improved survival in the same time periods, but the relative short followup and new treatment modalities may change this in the future.

## Figures and Tables

**Table 1:** Patient characteristics.

	Group 1	Group 2	*P* value
Males/females	29/23 (55.8%/44.2%)	61/48 (55.9%/44.0%)	1.00
Age at diagnosis (years)	62.0 (40–84)	62.0 (39–90)	0.54
Height, mean (cm)	173.48 (156–189)	172.40 (153–196)	0.538
Weight, mean (kg)	68.31 (40–100)	75.89 (42–123)	0.018
Duration of symptoms prior to diagnosis (months)	11.5 months (1–80)	6.0 months (0–168)	0.02
Carcinoid tumour symptoms at referral:			
(i) abdominal pain	34.7% (17/49)	25.2% (27/107)	0.25
(ii) diarrhea	55.1% (27/49)	39.3% (42/107)	0.82
(iii) flushing	30.6% (15/49)	33.6% (36/107)	0.85
(iv) bronchial obstruction	2.0% (1/49)	0.9% (1/107)	0.50

**Table 2:** Tumour characteristics.

	Group 1	Group 2	*P* value
Primary tumour size (mm)	30.7	25.2	0.60
Ki67 index	2% (1–7)	2% (1–80)	0.647
Metastases at referral:			
(i) liver metastases	55.8% (29/52)	44.0% (48/109)	0.18
(ii) carcinomatosis	28.8% (15/52)	9.2% (10/109)	0.02
(iii) lymph node metastases	25.0% (13/52)	43.1% (47/109)	0.036
(iv) bone metastases	5.8% (3/52)	2.8% (3/109)	0.389
(v) other metastases	5.8% (3/52)	7.3% (8/109)	1.0
Biochemical tumour markers (median (range)):			
(i) CgA level (pmol/L), (normal range: 30–130 pmol/L)	8709 (66–135500)	2381 (47–46500)	0.107
(ii) urine 5-HIAA (*μ*mol/day) (normal range 10–40 *μ*mol/day)	425 (20–3210)	212 (10–1590)	0.004

**Table 3:** Imaging modalities performed at referral.

	Group 1	Group 2	*P* value
CT scanning	38.5% (20/52)	78.9% (86/109)	0.000
Octreotide scintigraphy	78.8% (41/52)	74.3% (81/109)	0.530
MRI scanning	1.9% (1/52)	4.6% (5/109)	0.404
PET scanning	0.0% (0/52)	23.9% (26/109)	0.000

**Table 4:** Treatment.

	Group 1	Group 2	*P* value
Primary surgery:	80.8% (42/52)	75.2% (82/109)	0.549
(i) macroscopically radical	19.0% (8/42)	56.1% (46/82)	0.000
(ii) not macroscopically radical	81.0% (34/42)	43.9% (36/82)	
Interferon	65.4% (34/52)	42.2% (46/109)	0.007
Somatostatin analogues	73.1% (38/52)	66.1% (72/109)	0.470
Radionuclide treatment	9.6% (5/52)	13.8% (15/109)	0.610
Radiofrequency ablation (RFA)	1.9% (1/52)	15.6% (17/109)	0.008
Systemic chemotherapy	5.8% (3/52)	4.6% (5/109)	0.710
Temozolomide	1.9% (1/52)	1.8% (2/109)	1.000
Embolisation	0.0% (0/52)	8.3% (9/109)	0.030
New surgery:			
(i) none	100% (52/52)	89.9% (98/109)	0.000
(ii) small bowel resection	0.0% (0/52)	3.7% (4/109)	
(iii) lymph node resection	0.0% (0/52)	0.9% (1/109)	
(iv) liver resection	0.0% (0/52)	4.6% (5/109)	
(v) bone resection	0.0% (0/52)	0.9% (1/109)	

New surgery in total	0.0%	10.1%	
